# Vitamin D and thyroid disorders: a systematic review and Meta-analysis of observational studies

**DOI:** 10.1186/s12902-021-00831-5

**Published:** 2021-08-21

**Authors:** Sorour Taheriniya, Arman Arab, Amir Hadi, Abdulmannan Fadel, Gholamreza Askari

**Affiliations:** 1 Student Research Committee, Department of Community Nutrition, School of Nutrition and Food Science, Isfahan University of Medical Sciences, Isfahan, Iran; 2 Department of Community Nutrition, School of Nutrition and Food Science, Food Security Research Center, Isfahan University of Medical Sciences, Isfahan, Iran; 3 Department of Clinical Nutrition, School of Nutrition and Food Science, Food Security Research Center, Isfahan University of Medical Sciences, Isfahan, Iran; 4 School of Sport and Exercise Sciences, Faculty of Science, Liverpool John Moores University, Liverpool, UK

**Keywords:** Vitamin D, Thyroid disorders, Systematic review, meta-analysis

## Abstract

**Background:**

The contribution of vitamin D to thyroid disorders has received paramount attention; however, results are mixed. Hence, we designed a systematic review and meta-analysis to obtain a definitive conclusion.

**Methods:**

The search included PubMed, ISI Web of Science, Scopus, and Google Scholar databases up to March 2021 to collect available papers reporting the relationship between serum levels of vitamin D and thyroid disorders. The pooled effect was reported as weighted mean difference (WMD) and 95% confidence interval (CI).

**Results:**

Out of 6123 datasets, 42 were eligible to get into this systematic review and meta-analysis. Serum vitamin D was markedly lower in autoimmune thyroid diseases (AITD) (WMD − 3.1 ng/dl; 95% CI, − 5.57 to − 0.66; *P* = 0.013; I^2^ = 99.9%), Hashimoto’s thyroiditis (HT) (WMD − 6.05 ng/dl; 95% CI, − 8.35 to − 3.75; *P* < 0.001; I^2^ = 91.0%) and hypothyroidism patients (WMD − 13.43 ng/dl; 95% CI, − 26.04 to − 0.81; *P* = 0.03; I^2^ = 99.5%), but not in subjects with Graves’ disease (GD) (WMD − 4.14 ng/dl; 95% CI, − 8.46 to 0.17; *P* = 0.06; I^2^ = 97.5%).

**Conclusions:**

Our findings suggested lower vitamin D levels in patients with hypothyroidism, AITD, and HT compared to healthy subjects. However, the link between serum vitamin D and GD was only significant among subjects ≥40 years old.

## Background

Vitamin D is an essential fat-soluble nutrient with hormone-like activity [1, 2]. Vitamin D deficiency is among the most common health problems in the world in all age groups, even in low latitude countries with sufficient UV radiation or industrialized countries with a long history of vitamin D fortification strategies. Previous studies have indicated that about one billion people around the world have been diagnosed with vitamin D deficiency [3]. Recent evidence has shown the prevalence of vitamin D deficiency in both developed and developing countries, which in Europe is 13% and in the United States 19% [4–6]. Vitamin D deficiency has increased in recent years in Iran, and reports have revealed that the vitamin D deficiency rate is nearly 50% [2]. Risk factors for vitamin D deficiency include aging, female sex, winter season, obesity, malnutrition, lack of sun exposure, and dark skin pigmentation [7]. Accordingly, sufficient attention to vitamin D deficiency diagnosis is needed for treatment to decrease its undesirable effects on human health.

Over the last decades, numerous studies have indicated that low serum vitamin D levels are associated with a series of diseases such as high blood pressure, heart disease, diabetes, cancer, mood disorders, multiple sclerosis, and autoimmune diseases [8, 9]. This vitamin exerts its biological actions through nuclear vitamin D receptors (VDR) that reside in most human cells and tissues [10]. Hence, vitamin D plays its role through the regulation of gene expression in places where its receptors exist, such as the endocrine system [11].

The thyroid gland is one of the largest endocrine glands with many roles for homeostatic control, including growth, energy expenditure, and metabolism [12, 13]. Any thyroid disorder could result in a cluster of metabolic ailments [12–14]. Recent literature has illustrated the presumed association between serum vitamin D and thyroid diseases [15]. This association results from the presence of similar receptors for vitamin D and thyroid hormones, named steroid or nuclear hormone receptors. These shreds of evidence show the importance of the role of vitamin D in thyroid function and the association between vitamin D deficiency and thyroid diseases. However, some studies observed no significant relationship [16–18]. Therefore, we conducted a systematic review and meta-analysis to survey all observational studies regarding the association between serum vitamin D levels and thyroid disorders, including hypothyroidism, autoimmune thyroid disease (AITD), Hashimoto’s thyroiditis (HT), and Graves’ disease (GD) among the adult population.

## Methods

After registration on the Prospero database (CRD42020187237), this dataset was conceived pursuant to the Preferred Reporting Items for Systematic Reviews and Meta-analysis (PRISMA) statement [19].

### Search strategy

We performed a systematic literature search up to March 2021 in four databases, including PubMed, ISI Web of Science, Scopus, and Google Scholar to find papers reporting the relationship between serum levels of vitamin D and thyroid disorders. The terms used for the database search were “vitamin D” OR “25-hydroxyvitamin D” OR “1,25-dihydroxyvitamin D” OR “cholecalciferol” OR “ergocalciferol” OR “calcitriol” OR “vitamin D_3_” AND “hypothyroidism” OR “hypothyroid” OR “thyroid disorder” OR “triiodothyronine” OR “thyroxin” OR “thyroid stimulating hormone” OR “thyroid hormones” OR “hyperthyroidism” OR “hyperthyroid” OR “autoimmune thyroiditis” OR “anti-thyroid peroxidase” OR “Graves’^,^ disease”, OR “Hashimotoʼs thyroiditis”. The authors also screened the reference sections of the contained articles to spot any other eligible studies not captured via the online database search.

### Study selection

Before the screening process, all search results were exported into the EndNote X7 software (Thomson Corporation, Stamford, USA). Original observational human studies (case-control or cross-sectional) about the association between serum vitamin D and thyroid disorders with reported means, medians, or odds ratios (ORs), and the corresponding 95% confidence intervals (CIs) were included. Studies that recruited women during pregnancy or lactation, non-human datasets, reviews, case reports, editorials, poster abstracts, non-original, or irrelevant datasets were excluded. Two investigators were responsible for the selection process to lower the potential error (S. T & A. A). The difference of opinion was dealt with the cooperation of a third reviewer (G. A).

### Data extraction

The data of interest were extracted from each study using a predefined excel form in a duplicate and blinded manner. For each study, the extracted information was as follows: first author’s name, publication year, location, sample size, sex, age, body mass index (BMI), study design, vitamin D assay method, the season of sample collection, and population health status. Two independent reviewers conducted the processes of data extraction (S. T & A. A). The difference of opinion on a given study was dealt with via discussion, and if necessary, arbitration by a third reviewer (G. A).

### Study quality assessment

According to the Newcastle-Ottawa Quality Assessment Scale for observational studies [20], authors assessed the quality of the included studies on three domains: selection (5 points), comparability (2 points), and outcome (3 points). Scores of 7–10, 5–6, and 0–4 indicate high, moderate, and low-quality studies, respectively [21].

### Statistical analysis

Authors run a meta-analysis, including weighted mean difference (WMD) with 95% CIs plus a random effect model, to estimate the quantitative summary of the association between vitamin D and thyroid disorders, including hypothyroidism, AITD, HT, and GD. Mean ± standard deviation (SD) was extracted to estimate the pooled effects using WMD. For datasets with a standard error (SE), SD was determined using the following equation: SE × √n. Related equations were used to obtain the preferred data out of the median ± range or interquartile range [22]. If a study presented analyses stratified by certain key variables such as participants’ health status, stratified estimates were assumed to be independent of each other and included as a separate unit of observation in the meta-analysis. To assess the influence of each study on the stability of the meta-analysis results, the authors carried out a sensitivity analysis. Moreover, subgroup analysis based on age, gender, geographical population, study design, and population health status was executed whenever it was possible. STATA software, version 11.2 (Stata Corp, College Station, TX, USA) was used for data analysis with *P*-values < 0.05 considered as statistically significant. Heterogeneity was checked using the I^2^ index. I^2^ index equal to 25, 50, or 75% represents low, moderate, and high heterogeneity, respectively [23]. The funnel plots were visually inspected to detect any publication bias, and Egger’s and Begg’s tests defined the extent of funnel plot asymmetry [24].

## Results

### Search results

**Fig. 1:**
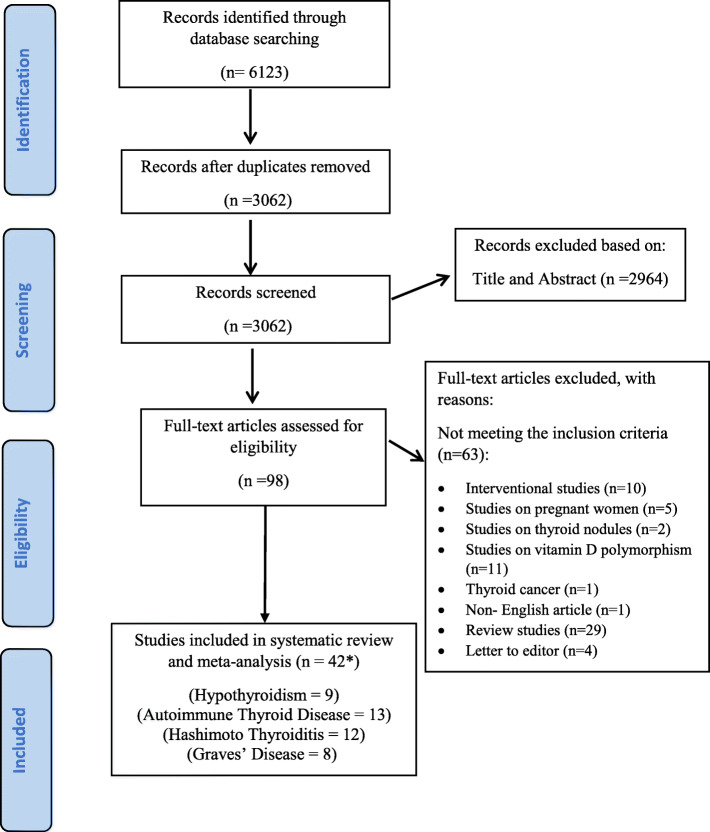
The flow diagram of study selection

Our initial search through databases identified a total of 6123 papers. With duplicates removed, the 3062 remaining articles were examined based on the review of the titles and abstracts by two independent reviewers. Authors retrieved and reviewed 98 articles based on the full text, and finally, 35 studies were eligible to get into the current study. Moreover, the works of Bouillon et al. [25], Unal et al. [26], Ma et al. [27], Ke et al. [28], Fawzy et al. [29], Toulis et al. [30], and Komisarenko et al. [31] were divided into two different studies. Therefore, a total of 42 eligible studies were included in the meta-analysis. The PRISMA flowchart briefly states the results of the study selection process (Fig. 1).

### Overview of included studies

A total of 35 studies (42 arms), including 28 case-control [17, 25–51] and 7 cross-sectional studies [52–58] with sample sizes varying from 38 to 6685 subjects were included in our systematic review. The selected studies were published between 1980 and 2018. Among these, 9 studies were from Turkey [26, 34, 36, 37, 39, 42, 43, 48, 49], 3 each from Saudi Arabia [17, 32, 35], Poland [38, 52, 59], China [27, 28, 51] and Korea [54, 55, 58], 2 each from Egypt [50, 60] and Italy [40, 57] and one each from Belgium [25], Canada [33], Brazil [44], Japan [47], India [45], Sweden [46], Thailand [53], Greece, United States [56], and Ukraine [31]. Among the 35 studies, 29 recruited both genders [25, 27–32, 34–41, 43–46, 48–56, 58], 4 only women [17, 42, 47, 57], and 2 studies did not report the gender [26, 33]. Eighteen studies mentioned BMI [27, 28, 30, 31, 33, 35, 36, 40, 45, 47, 50–53, 55–58] and 26 studies pointed to the time of year when samples were collected [17, 27–30, 32, 33, 36, 38–47, 49, 51, 53–55, 58, 61, 62]. All the included studies assessed the serum levels of 25(OH) D and none of them examined the dietary intake of vitamin D in participants. Additionally, among the selected studies, 6 [52–54, 56–58] were considered as high quality and the rest [17, 25–51, 55] had moderate quality. Table 1 gives an outline of the characteristics of the included studies.

**Table 1:** Characteristics of included studies

Author, Year	Location	Sample size (F/M)	Age (year)	BMI (kg/m^**2**^)	Study design	Population health status	Vit D assay method	Season of sample collection	Quality assessment score
**Bouillon et al., 1980 (a)**	Belgium	69/24	42	–	Case-control	Hypothyroid patients	RIA	–	Moderate
**Bouillon et al., 1980 (b)**	Belgium	77/27	39	–	Case-control	GD	RIA	–	Moderate
**Camurdan et al., 2012**	Turkey	126/26	12.05	–	Case-control	HT	HPLC	–	Low
**Yasuda et al., 2012**	Japan	72 F	40.8	21.75	Case-control	GD	Competitive protein binding assay	Winter, spring	Moderate
**Jyotsna et al., 2012**	India	124/36	36.37	22.05	Case-control	GD	RIA	All season	Moderate
**Chailurkit et al., 2012**	Thailand	1292/1290	55	23.3	Cross- sectional	AITD	HPLC	All season	High
**Bozkurt et. al., 2013**	Turkey	369/ 171	42.5	28.4	Case-control	HT	ELISA	Summer	Moderate
**Mackawy et al., 2013**	Saudi Arabia	35/25	46.38	–	Case-control	Hypothyroid patients	Spectrophotometry	Autumn, winter, spring	Moderate
**Aljohani et al., 2013**	Saudi Arabia	85/9	35.8	30.05	Case-control	Hypothyroid patients	ELISA	–	Moderate
**Fawzy et al., 2013 (a)**	Egypt	43/8	32.39	–	Case-control	Subclinical hypothyroid	CLIA	All season	Low
**Fawzy et al., 2013 (b)**	Egypt	44/12	31.66	–	Case-control	Hypothyroid patients	CLIA	All season	Low
**Ucar et al., 2014**	Turkey	62/15	74.95	–	Case-control	Subclinical hypothyroid	CLIA	–	Moderate
**Unal et al., 2014 (a)**	Turkey	378 F&M	44.6	–	Case-control	HT	CLIA	–	Low
**Unal et al., 2014 (b)**	Turkey	151 F&M	44.6	–	Case-control	GD	CLIA	–	Low
**Demir et al., 2014**	Turkey	24/14	12.95	–	Case-control	AITD	–	–	Moderate
**Zhang et al., 2014**	China	84/56	32.34	20.57	Case - control	GD	ELISA	Summer, autumn	Moderate
**Shin et al., 2014**	Korea	267/37	49.9	–	Cross sectional	AITD	RIA	All season	High
**Choi et al., 2014**	Korea	2793/3892	54.1	24.2	Cross-sectional	AITD	RIA	All season	Moderate
**Evliyaoglu et al., 2015**	Turkey	117/52	12.08	–	Case-control	HT	HPLC	Winter, spring, summer	Moderate
**Ma et al., 2015 (a)**	China	100/ 40	41.05	28.18	Case-control	HT	ECLA	Autumn, winter, spring	Moderate
**Ma et al., 2015 (b)**	China	97/43	41.01	28.18	Case-control	GD	ECLA	Autumn, winter, spring	Moderate
**Maciejewski et al., 2015**	Poland	84/10	47.62	–	Case-control	HT	ELISA	Spring	Moderate
**Toulis et al., 2015 (a)**	Greece	264 F&M	67.6	31.6	Case - control	AITD+T2DM	RIA	Winter, spring	Moderate
**Toulis et al., 2015 (b)**	Greece	234 F&M	72.2	30.6	Case - control	AITD+control group	RIA	Winter, spring	Moderate
**Muscogiuri et al., 2015**	Italy	50 F	26.8	28.1	Cross-sectional	AITD+PCOS	CLIA	Spring, summer	High
**Metwalley et al., 2015**	Egypt	88/24	14.32	18.8	Case-control	AITD	HPLC	–	Moderate
**Zhou et al., 2015**	USA	1076/930	37.8	26.68	Cross-sectional	AITD	–	–	High
**Sonmezgoz et al., 2016**	Turkey	76/68	11.12	–	Case-control	HT	CLIA	Autumn	Moderate
**Kim, 2016**	Korea	641/135	45.25	23.6	Cross-sectional	AITD	ECLIA	All season	High
**Giovinazzo et al, 2016**	Italy	175/25	41	26.5	Case-control	HT	HPLC	Autumn, winter, spring	Moderate
**Nalbant et al., 2017**	Turkey	453 F	40.3	–	Case-control	HT	ECLA	Spring, summer, autumn	Moderate
**Musa et al., 2017**	Saudi Arabia	116 F	35.7	–	Case-control	Hypothyroid patients	Spectrophotometry	Autumn, winter, spring	Moderate
**Ke et al., 2017 (a)**	China	65/47	38.68	22.57	Case-control	HT	ECLA	Autumn, winter	Moderate
**Ke et al., 2017 (b)**	China	61/41	38.13	22.42	Case-control	GD	ECLA	Autumn, winter	Moderate
**Lawnicka et al., 2017**	Poland	56/15	54.4	–	Case-control	HT	CLIA	Winter, spring	Moderate
**Planck et al., 2017**	Sweden	1167/1430	52.45	–	Case-control	GD	–	All season	Moderate
**Mirhosseini et al., 2017**	Canada	515 F&M	48	27.6	Case-control	Hypothyroid patients	HPLC	All season	Moderate
**Akdere G et al., 2018**	Turkey	66/94	28.3	–	Case-control	AITD+T1DM	ELISA	All season	Moderate
**Kmiec et al., 2018**	Poland	194/30	42	25.9	Cross-sectional	Hypothyroid patients	HPLC	Summer	High
**Komisarenko & Bobryk, 2018 (a)**	Ukraine	29/21	39.5	31.25	Case- control	AITD+T1DM	ELISA	–	Moderate
**Komisarenko & Bobryk, 2018 (b)**	Ukraine	31/19	57.25	37.25	Case- control	AITD+ T2DM	ELISA	–	Moderate
**Botelho et al., 2018**	Brazil	143/15	46.8	–	Case-control	HT	CLIA	Spring, summer	Low

F: Female, M: Male, BMI: Body Mass Index, RIA: Radio Immunoassay, CLIA: Chemiluminescence Immunoassay, HPLC: High Performance Liquid Chromatography, ALL: All seasons of the year, ELISA: Enzyme-Linked Immunosorbent Assay, HT: Hashimoto’s Thyroiditis, ECLA: Euglobulin Clot Lysis Assay, GD: Graves’ Disease, AITD: Autoimmune Thyroid Disease, ECLIA: Electrochemiluminescence Immunoassay, PCOS: Polycystic Ovary Syndrome, T1DM: Type 1 Diabetes Mellitus, T2DM: Type 2 Diabetes Mellitus

### Findings from the meta-analysis

#### Serum 25(OH) D and AITD

**Fig. 2:**
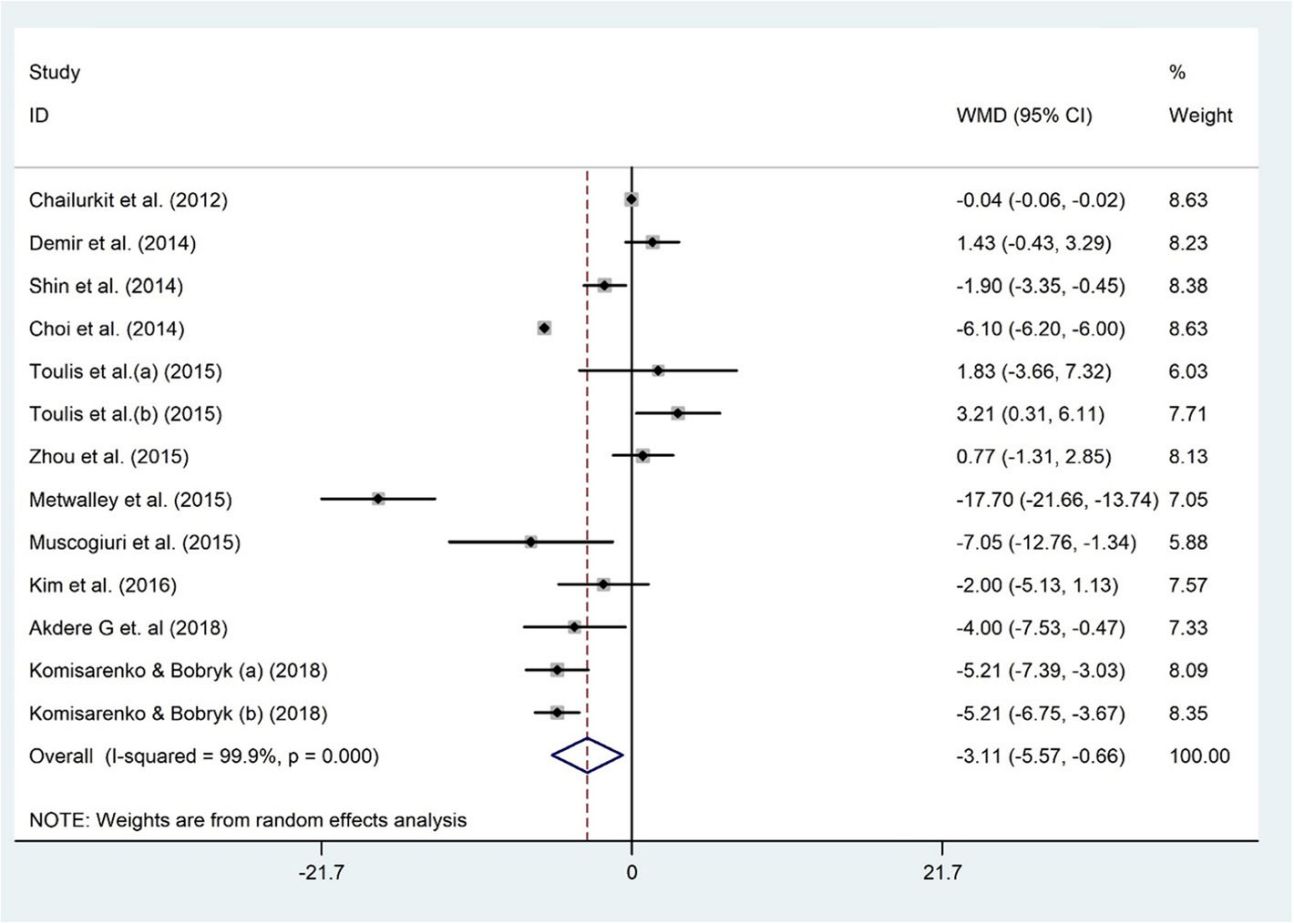
The association between the serum 25(OH) D level and AITD

Thirteen datasets comprising 12,916 participants, inspected the association between serum levels of 25(OH) D and AITD status among 1886 AITD diagnosed and 11,030 non-AITD individuals [30, 31, 48–50, 53–56, 58, 62]. Vitamin D was significantly associated with AITD status (WMD − 3.1 ng/dl; 95% CI, − 5.57 to − 0.66; *P* = 0.013) with significant heterogeneity (I^2^ = 99.9%, *P* < 0.001) (Fig. 2). In other words, patients with AITD showed significantly lower serum vitamin D levels compared to non-AITD subjects. In the metabolic disorders subgroup, vitamin D and AITD were related (WMD − 3.48 ng/dl; 95% CI, − 6.72 to − 0.24). However, no significant association between vitamin D and AITD was observed in the other subgroup (Table 2). Findings from the sensitivity analysis revealed that the exclusion of Metwalley et al. study from the analysis (WMD − 2.00 ng/dl; 95% CI, − 4.54 to 0.53) [50] modified the overall effect. No evidence of publication bias was documented (Begg’s test: *P* = 0.714, Egger’s test: *P* = 0.323).

**Table 2:** Subgroup analysis to assess the association between serum levels of vitamin D and thyroid disorders

Sub-grouped by	No. of studies	Effect size^**1**^	95% CI	I^**2**^ (%)	P for heterogeneity	P for between subgroup heterogeneity
**Hashimato**						
Age						**<0.001**
Adult	9	−5.69	−8.20, −3.18	89.6	<0.001	
Adolescent	3	−6.81	−11.70, −1.93	87.8	<0.001	
Geographical population						**0.05**
Turkey	6	−6.95	−11.15, −2.75	91.2	<0.001	
Other countries	6	−5.35	−9.13, −1.58	92	<0.001	
**Hypothyroid**						
Gender						**0.057**
Both sex	8	−15.43	−29.49, −1.37	99.6	<0.001	
Female	1	2.60	−1.38, 6.58	–	–	
Geographical population						**<0.001**
Asian	4	−12.34	−31.89, 7.20	99.3	<0.001	
Non-Asian	5	−14.31	−26.53, − 2.10	98.6	<0.001	
**Grave disease**						
Geographical population						**<0.001**
Asian	6	−1.74	−3.95, 0.46	80.2	<0.001	
Non-Asian	2	−7.10	−18.47, 4.25	99.3	<0.001	
Participants age						**<0.001**
≥ 40 years old	4	−8.79	−15.87, −1.72	98.1	<0.001	
< 40 years old	4	−0.54	−2.07, 0.98	54.3	0.08	
**AITD**						
Geographical population						**<0.001**
Asian	6	−2.08	−5.6, 1.43	100	<0.001	
Non-Asian	7	−4.13	−8.31, 0.05	93.9	<0.001	
Study design						**<0.001**
Case control	7	−3.65	−7.75, 0.44	94.4	<0.001	
Cross-sectional	6	−2.52	−6.10, 1.04	100	<0.001	
Participants health status						**<0.001**
With metabolic disorders	5	−3.48	− 6.72, −0.24	85.9	<0.001	
Without metabolic disorders	8	−2.87	−5.97, 0.23	99.9	<0.001	

^1^Calculated by Random-effects model

#### Serum 25(OH) D and GD

**Fig. 3:**
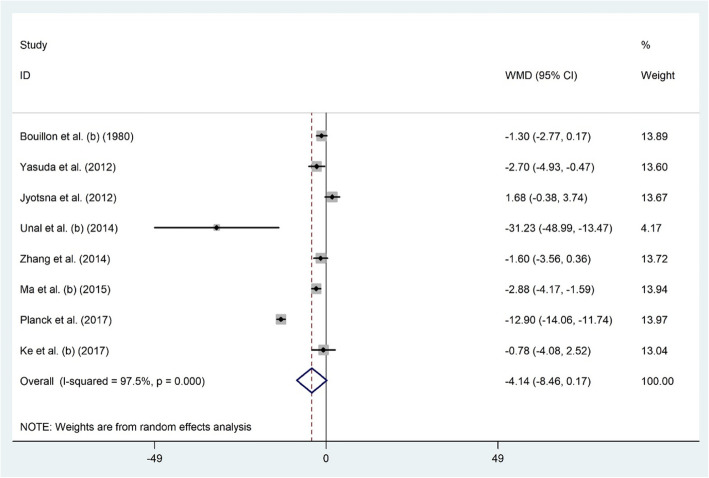
The association between the serum 25(OH) D level and GD

Eight citations addressed the link between serum levels of 25(OH) D and GD status among 604 GD diagnosed and 2827 non-GD individuals [25–28, 45–47, 51]. Overall, meta-analysis showed that serum vitamin D was not linked to GD (WMD − 4.14 ng/dl; 95% CI, − 8.46 to 0.17; *P* = 0.06) and heterogeneity was significant (I^2^ = 97.5%, *P* < 0.001) (Fig. 3). Subgroup analyses based on age and geographical area (Table 2) showed a significant association in studies recruiting ≥40 years-old subjects (WMD − 8.79 ng/dl; 95% CI, − 15.87 to − 1.72; I2 = 98.1). Jyotsna et al. study [45] influenced the meta-analysis results (WMD − 5.04 ng/dl; 95% CI, − 9.62 to − 0.46). Publication bias was not recognized (Begg’s test: *P* = 0.805, Egger’s test: *P* = 0.542).

#### Serum 25(OH) D and HT

**Fig. 4:**
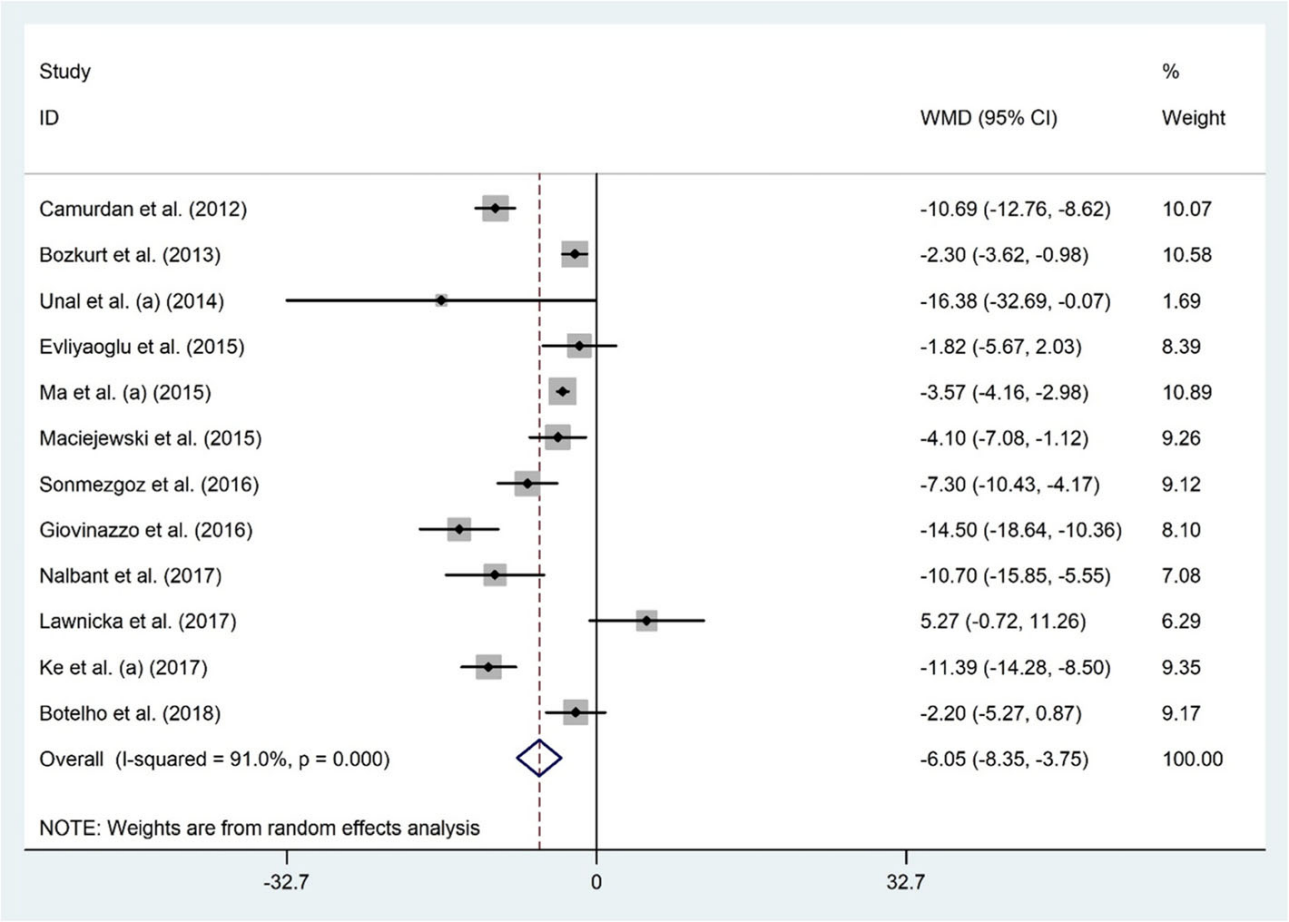
The association between the serum 25(OH) D level and HT

Twelve studies with 2440 participants examined the association between 25(OH) D and HT status among 1375 HT diagnosed and 1065 healthy subjects [26–28, 36–44]. The results of the meta-analysis indicated significantly lower levels of serum vitamin D among HT patients compared to healthy ones (WMD − 6.05 ng/dl; 95% CI, − 8.35 to − 3.75; *P* < 0.001) with significant heterogeneity (I^2^ = 91.0% *P* < 0.001) (Fig. 4). Subgroup analyses based on participants’ age and geographical area investigated the source of heterogeneity (Table 2). Keeping out the individual studies did not alter the overall meta-analysis results. Publication bias was not detected (Begg’s test: *P* = 0.784, Egger’s test: *P* = 0.175).

#### Serum 25(OH) D and hypothyroidism

**Fig. 5:**
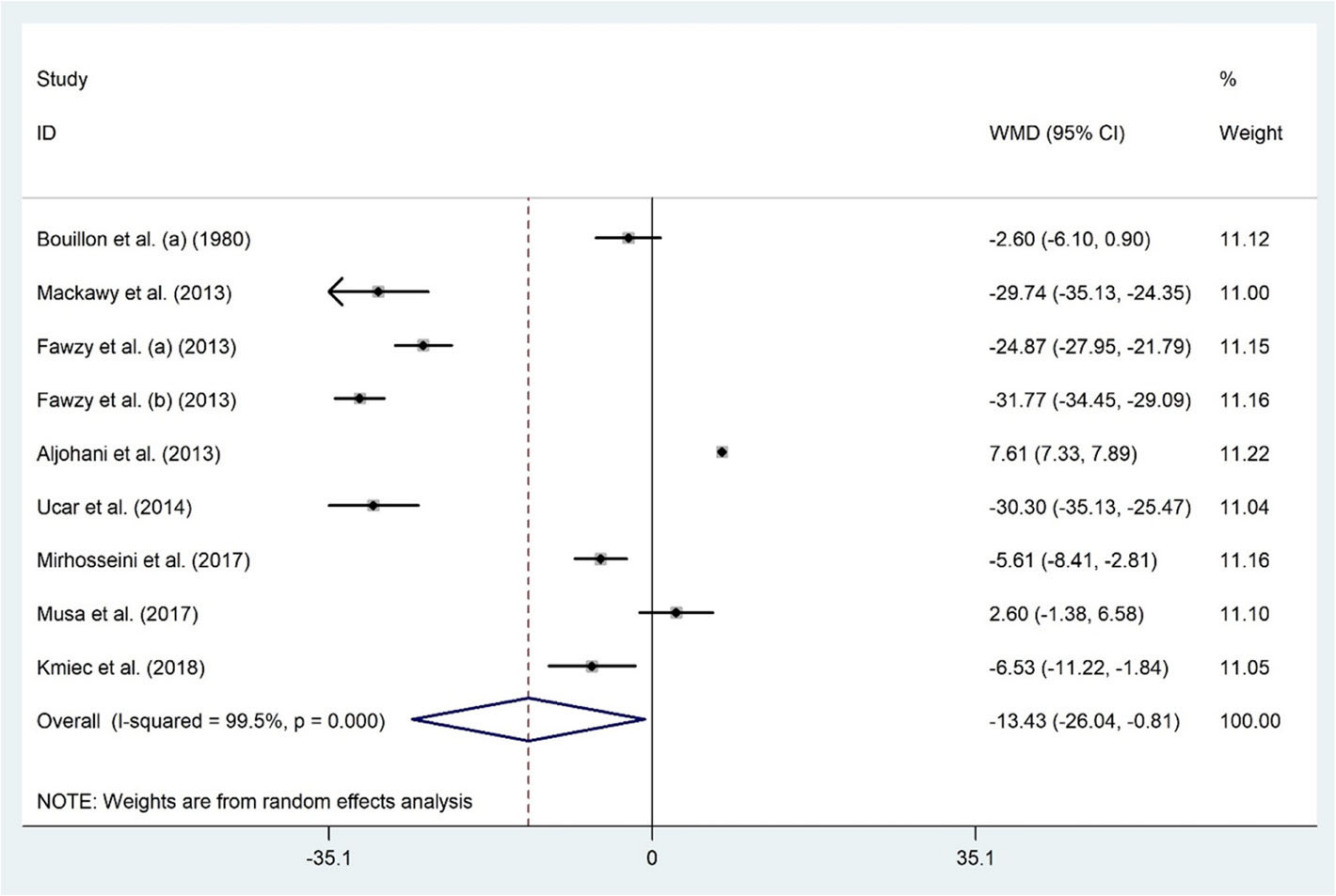
The association between the serum 25(OH) D level and hypothyroidism

Nine studies examined the relationship between serum levels of 25(OH) D and hypothyroidism among 372 hypothyroid patients and 802 healthy individuals [17, 25, 29, 32–35, 61]. Vitamin D and hypothyroidism status were significantly linked (WMD − 13.43 ng/dl; 95% CI, − 26.04 to − 0.81; *P* = 0.03); patients with hypothyroidism presented with lower serum vitamin D compared to healthy subjects. There was significant heterogeneity among the included studies (I^2^ = 99.5%, *P* < 0.001) (Fig. 5). Pursuant to subgroup analysis, vitamin D and hypothyroidism were associated only among studies recruiting both genders (WMD − 15.43 ng/dl; 95% CI, − 29.49 to − 1.37) and studies that included the non-Asian population (WMD − 14.31 ng/dl; 95% CI, − 26.53 to − 2.10) (Table 2). Keeping out a number of studies including Ucar et al. [34] (WMD − 11.33 ng/dl; 95% CI, − 22.76 to 0.08), Mackawy et al. [32] (WMD − 11.40 ng/dl; 95% CI, − 22.92 to 0.10), Mirhosseini et al. [33] (WMD − 14.41 ng/dl; 95% CI, − 29.01 to 0.18), Fawzy et al. (a) [29] (WMD − 11.98 ng/dl; 95% CI, − 24.84 to 0.86) and Fawzy et al. (b) [29] (WMD − 11.10 ng/dl; 95% CI, − 22.33 to 0.13) modified the overall effect. Due to evidence of publication bias (Begg’s test: *P* = 0.835, Egger’s test: *P* = 0.006), possible un-detected studies were tracked by trim and fill analysis, but this method could not add any dataset to our included ones.

## Discussion

As far as we know, this is the first comprehensive attempt to examine the association between serum levels of vitamin D and thyroid disorders in form of a systematic review and meta-analysis. It revealed that vitamin D was significantly associated with hypothyroidism, AITD, and HT. Furthermore, vitamin D was significantly lower among ≥40-year-old GD subjects. Some issues should be considered when elucidating the results. First of all, there were substantial inter-assay differences in the performance of commercially available kits for serum vitamin D assay [63]. This notion may impact the results and play a considerable role as a heterogeneity factor. Also, seasonal variation in serum vitamin D should be kept in mind when interpreting the results [64]. This fact has been excused in some papers [25, 26, 31, 34, 35, 37, 46, 48, 50, 56]. Inconsistencies in terms of the season of sample collection could also influence our final results. Besides, it has been proven that the bioavailability of vitamin D diminishes among overweight/obese individuals [65]; however, most of the included articles disregarded BMI as a confounding factor and probable source of heterogeneity [17, 18, 25, 26, 29, 32, 34, 37–39, 41–44, 46, 48, 49, 54].

Pursuant to previous reports, the role of vitamin D in immunity is through producing anti-inflammatory and immune-regulatory markers via VDR expression in the nucleus of cells [66]. VDR is involved in cellular immunity function by stimulating innate and adaptive immune responses [66]. Polymorphisms of the VDR are related to the predisposition of people to thyroid disorders such as hypothyroidism [12, 67]. VDR modulates the effects of vitamin D as its specific and intracellular receptor. Polymorphisms of the VDR gene may decrease vitamin D activity [12]. Through a meta-analysis, Wang et al., reported a significant link between VDR gene polymorphisms and autoimmune thyroid disorders in diverse ethnic groups [66]. Vitamin D has hindering effects on the production of inflammatory cytokines such as interleukin (IL)-1, IL-6, IL-8, IL-12, and tumor necrosis factor (TNF)-α [68, 69]. Also, it suppresses dendritic cell differentiation and maturation via a reduced expression of the major histocompatibility complex (MHC) class II molecules, co-stimulatory molecules, and IL-12 [68, 69]. Additionally, vitamin D makes the induction of T regulatory cells simpler to diminish T cell-dependent immune responses in autoimmune diseases [68, 69]. T and B-cells reaction to thyroid antigens in genetically-vulnerable subjects lead to HT [42]. Based on previous studies, serum levels of 25-OH D3 < 20 ng/mL can exacerbate positive thyroid autoantibodies such as anti-thyroid peroxidase (TPOAb) and anti-thyroglobulin (TgAb) [70–72]. Additionally, high serum levels of calcium and phosphorous and low levels of circulating parathyroid hormone may inhibit renal 25(OH) D1-α hydroxylase function [17]. Thus, due to lower production of 1,25 (OH)2 D, serum 24,25 (OH) D and thyroid hormone levels will be higher [17]. Overall, vitamin D could contribute to the prevention or correction of hypothyroidism and improvement of thyroid function [17].

The limitations of this study are as follows. There was significant heterogeneity in our study that may have affected the results and diminished the generalizability of the outcomes. The probable sources of heterogeneity might be differences in age, gender, BMI, study design, vitamin D assay methods and kits, the season of sample collection, geographical variation, and quality of the studies. Moreover, the nature of cross-sectional studies makes it impossible to draw a causal link between variables. Furthermore, there was no information regarding dietary vitamin D intake that could have affected serum levels of vitamin D.

## Conclusion

In the present systematic review and meta-analysis, vitamin D levels were significantly lower in hypothyroidism, AITD, and HT patients compared to healthy people. However, there was no significant association between serum vitamin D and GD, except among subjects ≥40 years old. The effect sizes of the included studies showed significant heterogeneity which might affect the interpretation of results. Further well-designed prospective cohort studies and clinical trials are needed for a better understanding of the relationship between vitamin D and thyroid disorders.

## Data Availability

The data that support the findings of this study are available from the corresponding author upon reasonable request.

## Abbreviations

Vitamin D: Vitamin D
VDR: Vitamin D Receptor
AITD: Autoimmune thyroid disease
HT: Hashimotoʼs thyroiditis
GD: Graves’ disease
PRISMA: Preferred reporting items for systematic reviews and meta-analysis
OR: Odds ratio
CI: Confidence intervals
BMI: Body mass index
WMD: Weighted mean difference
SD: Standard deviation
SE: Standard error
IL: Interleukin
TNF: Tumor necrosis factor
MHC: Major Histocompatibility Complex
TPOAb: Anti-thyroid peroxidase Antibody
TgAb: Anti-thyroglobine Antibody
